# Randomized study of intravesical pirarubicin chemotherapy with low and intermediate-risk nonmuscle-invasive bladder cancer in Japan: Comparison of a single immediate postoperative intravesical instillation with short-term adjuvant intravesical instillations after transurethral resection

**DOI:** 10.1097/MD.0000000000012740

**Published:** 2018-10-19

**Authors:** Yoshio Naya, Kazuya Mikami, Natsuki Takaha, Yuuta Inoue, Atsuko Fujihara, Motohiro Kanazawa, Hiroyuki Nakanishi, Hiroaki Miyashita, Osamu Ukimura

**Affiliations:** a Department of Urology, Meiji University of Integrative Medicine, Nantan; b Department Urology, Nagahama City Kohoku Hospital, Nagahama; c Department of Urology, Kyoto First Red-Cross Hospital; d Department of Urology, Kyoto Prefectural University of Medicine, Kyoto; e Department of Urology, Kyoto Chubu Medical Center, Nantan; f Department of Urology, Omihachiman Community Medical Center, Omihachiman, Japan.

**Keywords:** intravesical chemotherapy, pirarubicin, survival rate, urinary bladder neoplasms

## Abstract

**Purpose:**

The objective of this study was to evaluate the efficacy, defined by the 3-year tumor recurrence-free survival rate, of intravesical chemotherapy using pirarubicin (THP) in patients with low or intermediate-risk nonmuscle-invasive bladder cancer (NMIBC).

**Patients and methods:**

Between October 2010 and January 2015, 206 patients were enrolled, and finally 113 were randomized to receive either a single immediate postoperative intravesical instillation of THP (30 mg) (Group A), or 8 additional weekly intravesical instillations of THP (30 mg) after a single postoperative instillation (Group B). The patients were examined by performing cystoscopy and urine cytology every 3 months after transurethral resection to determine bladder tumor recurrence. The primary endpoint was 3-year-recurrence-free survival rate.

**Results:**

All 113 patients were bacillus Calmette–Guérin (BCG)-naïve. The 3-year recurrence free survival rate was 63.7% for Group A and 85.3% for Group B (log-rank test, *P* = .0070). In patients with intermediate recurrence risk, the 3-year recurrence-free survival rate was 63.4% in Group A and 86.1% in Group B (log-rank test, *P* = .0036). Cox regression analysis revealed that only additional instillation of THP was a significant independent factor for recurrence-free rate in patients with intermediate risk. No patient with progression was noted during this period. Frequent adverse effects (AEs) were frequent urination and micturition pain, and no severe AEs (Grade 3 or more) occurred.

**Conclusion:**

Additional instillation of THP (30 mg) weekly for 8 weeks reduced the risk of tumor recurrence without severe AEs in BCG-naïve NMIBC patients with intermediate risk.

## 1 Introduction

Several guidelines recommend a single immediate postoperative intravesical instillation of chemotherapy for standard and sufficient treatment of low-risk nonmuscle-invasive bladder cancer (NMIBC).^[1–3]^ A recent meta-analysis showed that a single instillation reduced the overall risk of recurrence by 35% and the 5-year recurrence rate from 58.8% to 44.8%.^[4]^

For patients with an intermediate-risk for NMIBC, both intravesical bacillus Calmette–Guérin (BCG) therapy and chemotherapy are recommended as viable options as per several guidelines.^[1–3]^ Additional adjuvant intravesical chemotherapy after surgical intervention (SI) was considered to improve recurrence-free survival in patients with intermediate-risk. A large meta-analysis of 3703 patients from 11 randomized trials showed a highly significant 44% reduction in the odds of recurrence at 1 year in favor of chemotherapy over transurethral resection of bladder tumors (TURs) alone, consistent with the European Association of Urology (EAU) guidelines.^[4]^ The optimal schedule and duration of intravesical chemotherapy is controversial, but should focus on eradicating residual disease after TUR and preventing late recurrences.^[5]^ We conducted a randomized, prospective study comparing single immediate postoperative intravesical chemotherapy with short-term adjuvant intravesical chemotherapy after TUR in NMIBC patients with low or intermediate risk. The objective was to evaluate the efficacy, which was defined by the 3-year recurrence-free survival rate, of intravesical chemotherapy using pirarubicin (THP) for patients with low or intermediate risk.

## 2 Materials and methods

**Figure 1:**
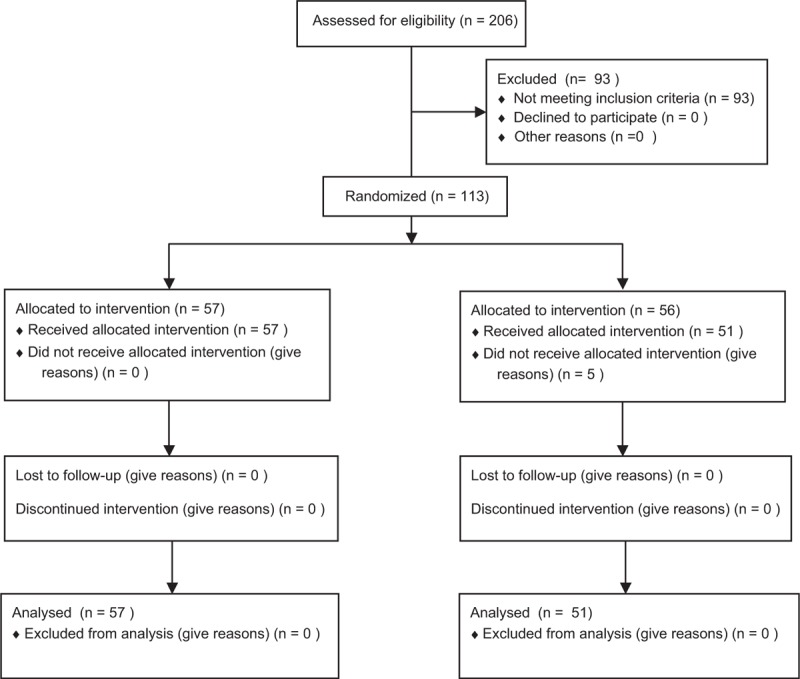
Consolidated standards-of-reporting-trials diagram. ITT = intention to treat, PPS = per protocol set.

This study was a multicenter, prospective, randomized controlled trial, and was approved by the ethics committees of the Kyoto Prefectural University of Medicine in September 2010 (RBMR-C-815-1). The study was registered as UMIN000004861. Informed consent was obtained from all patients before study enrollment. The study design is shown in Fig. 1. The recurrence risk was stratified using EAU guidelines on nonmuscle-invasive urothelial carcinoma of the bladder, as per the 2009 update. The patients with a recurrence-risk score of 0 were stratified to a low-risk group and those with scores ranging from 1 to 9 were stratified to an intermediate-risk group. Between October 2010 and January 2015, a total of 206 patients from Kyoto Prefectural University of Medicine, Kyoto First Red-Cross Hospital, Matsushita Memorial Hospital, Nantan Hospital, and Ohmihachiman City Hospital scheduled to undergo TUR were enrolled in this study. Of these, 93 were excluded after pathological confirmation, and the remaining 113 patients were randomized by computer-aided randomization procedures to receive either a single immediate postoperative intravesical instillation of 30 mg of THP (Group A) only or additional intravesical chemotherapy of 30 mg of THP weekly for 8 weeks after a single postoperative instillation (Group B). The additional instillation was initiated 1 or 2 weeks after TUR, contingent on a confirmation of normalization by urine examination. Based on the results of a prospective study from Osaka University,^[5]^ we performed additional intravesical chemotherapy with 30 mg of THP administered weekly for 8 weeks. Study inclusion and exclusion criteria are shown in Table 1. Patients with muscle-invasive bladder cancer, grade 3 urothelial carcinoma and/or carcinoma in situ (CIS) were excluded.

**Table 1:**
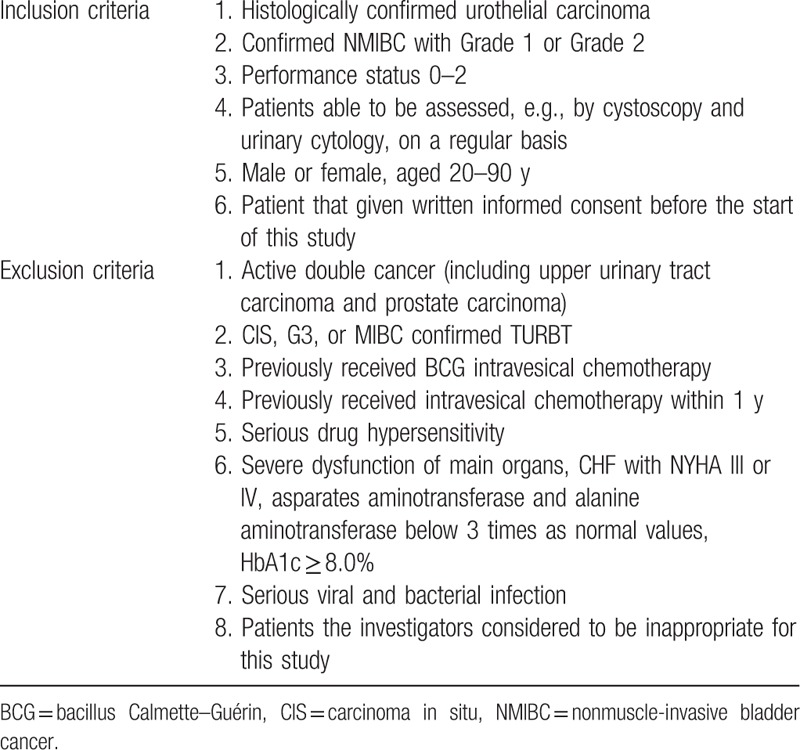
Study inclusion and exclusion criteria.

Patients were screened for bladder tumor recurrence by cystoscopic examinations and urine cytological examinations every 3 months for 3 years after TUR, and every 6 months thereafter (Fig. 1). The primary endpoint was the 3-year-recurrence-free survival rate and the secondary endpoint was occurrence of adverse effects (AEs).

### 2.1 Statistical analysis

A previous clinical trial reported that the 2- or 3-year recurrence-free survival rates in patients receiving a single immediate postoperative intravesical chemotherapy and in those receiving additional intravesical instillation therapy were 60% and 75%, respectively.^[6–9]^ Tolley et al^[8]^ as a comparison test which added a single-shreds note and subsequent maintenance therapy, 2-year nonrecurrence rate was a single-dose group 58% Maintenance Therapy Group 69%. Okamura et al^[9]^ compared the short-term maintenance therapy and the prolonged maintenance therapy, each 2-year nonrecurrence rate was 75% to 77%. Based on these, a sample size was calculated to test the null hypothesis of superiority in 3-year recurrence free survival probabilities after additional intravesical chemotherapy, compared to that after a single postoperative injection (73% of additional intravesical chemotherapy vs 60% of a single postoperative injection, respectively), with the registration period 2 years, tracking 3 years α = 0.1, to determine the number of required cases as β = 0.2. Based on our analysis, 127 patients were required in each arm, and we planned to enroll 130 patients in each arm. A total of 206 patients were enrolled initially, and finally, 113 were registered after the exclusion of those who did not meet the eligibility criteria. There were no patients who underwent previous BCG therapy in this study. The 113 patients were randomized by computer-aided randomization procedures (Latin390 using Y.N.) to receive either a single immediate postoperative intravesical instillation of 30 mg of THP (Group A) only or 8 additional intravesical chemotherapy instillations of 30 mg of THP weekly, starting 1 or 2 weeks after a single postoperative instillation (Group B) (Fig. 1). The primary endpoint was analyzed in intent-to-treat subjects. Kaplan–Meier curves were constructed for each study arm. The null hypothesis was tested using the log-rank test. Cox regression analysis was also performed for recurrence-free survival rates. Statistical analysis was performed using the SAS software (SAS Institute, Inc., Cary).

## 3 Results

**Figure 2:**
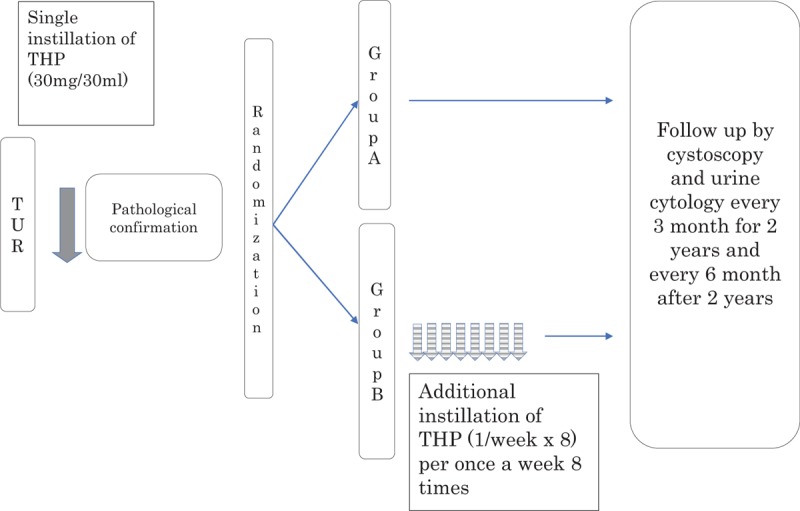
Schema of study design. TUR = transurethral resection of bladder tumor.

Of 206 initially enrolled patients, 93 were excluded. The reasons for exclusion included grade 3 cancer or CIS (n = 57), no pathological evidence of malignancy (n = 16), pT2 stage disease (n = 4), patient's wish (n = 4), prior intravesical chemotherapy within 1 year (n = 2), perforation at TUR (n = 2), postoperative urinary tract infection (n = 2), and others (n = 6). The remaining 113 patients were randomized to Group A (n = 57) or Group B (n = 56). In Group B, 5 patients did not complete all the 8 scheduled instillations due to AEs (Fig. 2). Patient profiles are summarized in Table 2. All patients were BCG-naïve. Of 113 patients, only 13 patients had low recurrence-risk. There were no significant differences in background characteristics between Group A and B. In Group B, median time to start additional instillation after TUR was 17 ± 9 days (6–38).

**Table 2:**
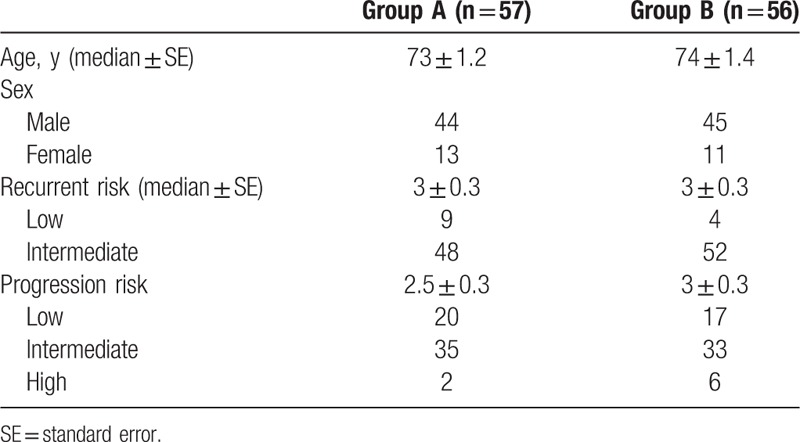
Patient characteristics.

**Figure 3:**
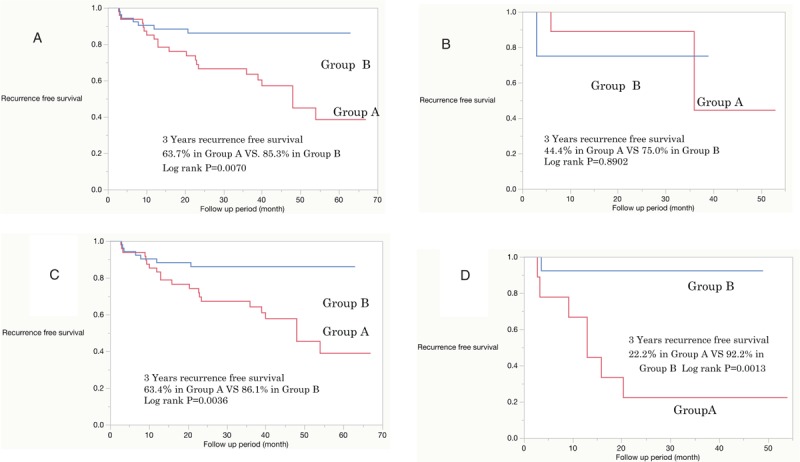
Recurrence-free survival curves after intravesical instillation of pirarubicin (THP). (A) Overall, (B) low risk, (C) intermediate risk, (D) recurrent risk score between 5 and 9.

The median follow-up period was 36 months. The 3-year recurrence-free survival rate was 69.3% for Group A and 85.3% for Group B (log-rank test, *P* < .01) (Fig. 3A). In patients with low risk, the 3-year recurrence-free survival was 44.4% in Group A and 75.0% in Group B (log-rank test, *P* ≥ .1). The recurrence-free survival at 30 months was 88.9% in Group A and 75.0% in Group B (Fig. 3B). In patients with intermediate recurrence risk, the 3-year recurrence-free survival was 63.4% in Group A and 86.1% in Group B (log-rank test, *P* < .01) (Fig. 3C). In patients with recurrence scores between 5 and 9, the 3-year recurrence-free survival was 92.3% in Group B and 22.2% in Group A (log-rank test, *P* < .01) (Fig. 3D). Cox regression analysis revealed that only additional instillation of THP was an independent factor for tumor recurrence in patients with intermediate risk (Table 3). Disease progression was not noted during this period in any patient.

**Table 3:**
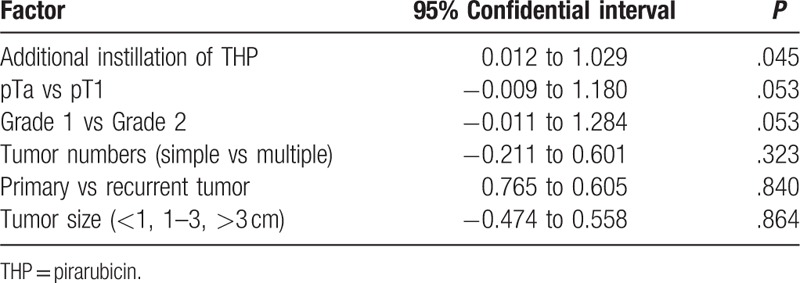
Multivariate Cox regression analysis for tumor recurrence in patients with intermediate risk.

The AEs were scored according to the Common Terminology Criteria for Adverse Events (CTCAE) Version 4.0 during the treatment period (Table 4). Commonly reported AEs were frequent urination and pain on urination. The incidence of AEs was significantly higher in Group B than in Group A (*P* < .001, Wilcoxon rank sum test). In Group B, the percentage of patients who completed all 8 additional instillations of THP was 91.1% (51 of 56). Therapy could not be completed as scheduled in 5 patients due to AEs such as frequent urination (3 patients with Grade 2 and 1 patient with Grade 1) and pain on urination (1 patient with Grade 1). Severe AEs (Grade 3 or above) did not occur in any of the patients.

**Table 4:**
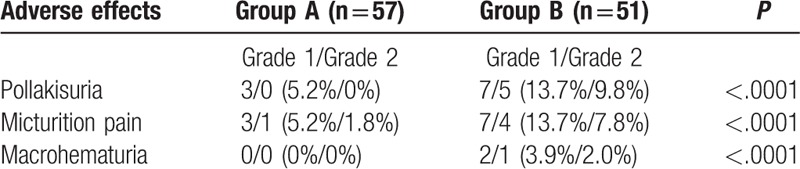
Grades and percentages of adverse effects in each arm.

## 4 Discussion and conclusion

In low-risk NMIBC patients, a single immediate postoperative intravesical instillation of chemotherapy is standard and sufficient treatment.^[1–3]^ In this study, the single instillation regimen was effective for 30 months and additional instillations did not contribute to the prevention of tumor recurrence. Our results were in agreement with a previous study by Sylvester et al.^[4]^ In this study, most patients displayed intermediate recurrence-risk. Guidelines issued by the National Comprehensive Cancer Network, EAU, or the Japanese Urological Association recommend intravesical BCG therapy or intravesical chemotherapy in patients with NMIBC who have an intermediate recurrence risk.^[1–3]^ Intravesical chemotherapy is recognized as an efficient therapeutic option for the prevention of recurrence in low or intermediate recurrence-risk patients, even though the there is no agreement regarding a standardized protocol for intravesical chemotherapy.^[7]^ Kawamura et al^[10]^ conducted a 3-week randomized study comparing THP versus adriamycin (ADM) instillation in superficial bladder cancer. They concluded that a half-dose of THP was equivalent to that of one standard dose of ADM. In this study, additional weekly (8 times) intravesical chemotherapy using 30 mg of THP was more effective compared to the postoperative single immediate intravesical chemotherapy, without severe complications. Li et al^[11]^ reported that the postoperative single immediate intravesical chemotherapy and additional 30 mg/50 mL of THP instillation once a week for 8 weeks was more effective compared to THP instillation once a week for 8 weeks without a single immediate postoperative treatment of intravesical chemotherapy. In this study, additional intravesical chemotherapy was the only independent prognostic factor for tumor recurrence in patients with intermediate risk. It is noteworthy that a significantly high 3-year recurrence-free survival rate was observed in Group B with a recurrence score between 5 and 9 compared to that in Group A. In this study, all patients were BCG-naïve. Therefore, 8 additional weekly instillations of intravesical chemotherapy using THP might be a useful option in BCG-naïve patients with NMIBC with intermediate recurrence-risk. In addition, patients with a recurrence score between 1 and 4 were comparable to those with low risk in this study.

A limitation of this study is that a small number of patients were enrolled and hence statistical analysis could not be performed. To confirm these results, future prospective studies with a larger sample size are needed.

Approximately 80% of patients who underwent BCG therapy had AEs of any type.^[12]^ Okamura et al^[9]^ reported that approximately 10% of patients had local toxicity of intravesical THP chemotherapy. Although the incidence of AEs in patients who underwent THP maintenance therapy was higher than the incidence in patients who underwent a single instillation, the incidence of AEs due to intravesical instillation of THP was lower compared to AEs associated with BCG therapy. In addition, severe AEs were not observed in any of the patients who underwent additional intravesical instillation of THP for 8 weeks.

The aim of this study was to test the efficacy of additional intravesical instillations of THP in patients with NMIBC. Our data clearly show that 8 additional weekly instillations of intravesical chemotherapy using THP after a single immediate intravesical chemotherapy instillation post-TUR in BCG-naïve patients with intermediate recurrence risk confer survival and recurrence benefits.

In conclusion, this prospective randomized study demonstrated the efficacy of additional intravesical chemotherapy using THP in BCG-naïve NMIBC patients with intermediate recurrence-risk without severe complications. Additional intravesical THP chemotherapy is a viable therapy option for NMIBC patients with intermediate risk and can improve survival outcomes.

## Acknowledgments

We would like to thank the KPUM oncology Group (Collaborators of the KPUM Oncology Group: M. Maegawa, M. Kojima, H. Miyashita, A. Fujito, T. Kitamori, K. Yoneda, Y. Ito, M. Nakamura, T. Iwata, M. Inaba, N. Kanemitsu, K. Masuda, N. Sato, S. Ushijima, N. Hirahara). We also would like to thank Editage (www.editage.jp) for English language editing.

## Author contributions

**Conceptualization:** Yoshio Naya, Kazuya Mikami.

**Data curation:** Natsuki Takaha, Motohiro Kanazawa, Hiroaki Miyashita.

**Formal analysis:** Yuta Inoue, Atsuko Fujihara.

**Investigation:** Yoshio Naya.

**Methodology:** Hiroyuki Nakanishi.

**Supervision:** Osamu Ukimura.

**Writing – original draft:** Yoshio Naya.

**Writing – review & editing:** Yoshio Naya.
